# Instagram use is linked to increased symptoms of orthorexia nervosa

**DOI:** 10.1007/s40519-017-0364-2

**Published:** 2017-03-01

**Authors:** Pixie G. Turner, Carmen E. Lefevre

**Affiliations:** 1 Division of Medicine, UCL, London, WC1E 6BT UK; 2 Centre for Behaviour Change, Department of Clinical, Education and Health Psychology, UCL, London, WC1E 7HB UK

**Keywords:** Orthorexia nervosa, Eating disorder, Social media, Instagram

## Abstract

**Purpose:**

Social media use is ever increasing amongst young adults and has previously been shown to have negative effects on body image, depression, social comparison, and disordered eating. One eating disorder of interest in this context is orthorexia nervosa, an obsession with eating healthily. High orthorexia nervosa prevalence has been found in populations who take an active interest in their health and body and is frequently comorbid with anorexia nervosa. Here, we investigate links between social media use, in particularly Instagram and orthorexia nervosa symptoms.

**Methods:**

We conducted an online survey of social media users (*N* = 680) following health food accounts. We assessed their social media use, eating behaviours, and orthorexia nervosa symptoms using the ORTO-15 inventory.

**Results:**

Higher Instagram use was associated with a greater tendency towards orthorexia nervosa, with no other social media channel having this effect. In exploratory analyses Twitter showed a small positive association with orthorexia symptoms. BMI and age had no association with orthorexia nervosa. The prevalence of orthorexia nervosa among the study population was 49%, which is significantly higher than the general population (<1%).

**Conclusions:**

Our results suggest that the healthy eating community on Instagram has a high prevalence of orthorexia symptoms, with higher Instagram use being linked to increased symptoms. These findings highlight the implications social media can have on psychological wellbeing, and the influence social media ‘celebrities’ may have over hundreds of thousands of individuals. These results may also have clinical implications for eating disorder development and recovery.

## Introduction

Social media use is constantly increasing, with now 90% of UK young adults (aged 16–34 years) accessing social media platforms [1]. As such it is important to better understand the effects that social media use may have on mental wellbeing. One example is the social media based healthy eating community, which has recently grown in popularity [2]. While overall this movement has been positive, with members striving to eat more fruits and vegetables and fewer processed foods, there is a growing concern around it triggering negative behaviours and eating disorders [3]. One eating disorder of interest in this context is orthorexia nervosa (ON), an obsession with eating healthily [4]. Previous work around similar disorders suggests that social media use may contribute to an ‘echo-chamber’ effect, where users perceive their values and world-views to be more common than they actually are, due to selectively viewing contributions of other, similarly minded people [5]. In light of this effect, here we investigated whether time spent on social media is associated with symptoms of ON. We focus on Instagram, as it is most prevalent in the healthy-eating movement [3] but also assess other social media channels in an exploratory analysis.

Instagram, launched in 2010, is an online social networking service that is currently used by 53% of American young adults (aged 18–29 years) with internet access [6]. The platform enables users to post pictures and videos to their profiles, add a caption, use hashtags (# symbol) to describe the photos, and tag other users (@ symbol). Users can follow any number of accounts and view a steady stream of content posted by the users they follow where they can “like” or “comment” on posts. Instagram suggests new accounts to follow based on the content that the user is already exposed to. Posts are dominated by the content of the images rather than any captions or comments. In June 2016 Instagram had 500 million registered users worldwide [7], making it the third biggest social media platform after Facebook and Tumblr. A US poll showed the average user spends 21.2 min on Instagram each day, with the 18–29 age group spending the most time at 30 min [8].

The hashtag #food is one of the top 25 most popular hashtags on Instagram.^1^ A more detailed analysis of Instagram photo content found it to be one of eight popular categories along with self-portraits (or “selfies”), friends, activities, captioned photos, gadgets, fashion, and pets [9], suggesting the importance of Instagram for the sharing of food-related content. Healthy food posts tend to receive more support from users than less healthy images [10], indicating a positive attitude towards healthy foods and healthy eating. Research also suggests that social media is used to inform actual food choices, with 54% of consumers using social media to discover and share food experiences, and 42% using social media to seek advice about food [11]. Documentation, surveillance, and creativity have all been identified as motivations for Instagram use [12, 13], which corresponds well with the sharing of food images—both as a food diary (documentation/creativity) and searching for recipes and inspiration (surveillance).

Instagram and social media more broadly have been associated with mental health problems. For example, social media use has been associated with higher levels of depression in young adults [14], as well as eating disorders and related behaviours [15]. For example, adolescents who view health and fitness-related content on social media are more likely to have an eating disorder [15]. In patients with AN, extensive Facebook use is associated with greater levels of symptoms [16], and viewing pro-anorexia websites worsened psychological symptoms in an experimental study when compared to control participants who viewed neutral websites [17]. More frequent Instagram use has been directly associated with greater levels of depressive symptoms, and following a greater number of strangers is associated with greater levels of negative social comparison [18]. An analysis of the #fitspiration tag on Instagram, a tag used to denote images intended to inspire people to become fit and healthy, found that the majority of images of women showed a thin and toned body type with objectifying elements, which could have negative effects on body image and self-esteem [19]. Moreover, researchers have pointed out the key role of social comparison in body image disturbance [20]. However, image-based platforms such as Instagram have also been shown to confer a significant decrease in self-reported loneliness to users, whereas text-based platforms such as Twitter do not [21]. Taken together, while there is some evidence for negative links between social media use and mental health, the picture is far from clear and social media may indeed also have positive or protective qualities in certain circumstances.

Recently there has been much media coverage on the role of social media, particularly Instagram, in the ‘healthy eating movement’ in the UK [2, 22], despite a lack of current academic literature on the subject. Pioneers of this movement have a powerful social media presence, particularly on Instagram, reaching and influencing hundreds of thousands of people, despite often having no formal training in health sciences or nutrition. Because Instagram is an image-based platform, users may be more likely to follow advice or imitate the diets of Instagram ‘celebrities’ as they feel a more personal connection than they would on a text-based platform. Although often not based on scientific evidence, individuals are encouraged to cut out various food groups from their diets, potentially leading to an unbalanced diet and deficiencies. Moreover, this advice may encourage psychological problems around food, and in some cases, lead to eating disorders such as anorexia nervosa (AN) [3] or ON.

Orthorexia nervosa is defined as an unhealthy obsession with eating healthy food. The term is derived from the Greek “orthos,” meaning “correct”, and was coined by Steven Bratman [4]. Despite not appearing in the Diagnostic and Statistical Manual of Mental Disorders (DSM) [23], ON has sparked a body of research. The proposed diagnostic criteria for ON [24] include obsessive focus on healthy eating, food anxiety, and dietary restrictions, with these behaviours causing clinical impairments. There is some overlap between ON, AN and obsessive compulsive disorder (OCD) [25]. Both AN and ON share traits of perfectionism, cognitive rigidity, and guilt over food transgressions, while OCD and ON share intrusive thoughts and ritualised food preparation. However, while AN patients are preoccupied with the quantity of food, ON patients are preoccupied with the quality of food.

Orthorexia symptoms are associated with healthy lifestyle choices such as eating more fruit and vegetables, eating less white cereals, shopping in health food stores, exercise, and reduced alcohol consumption [26]. But ON is also associated with significant dietary restrictions, malnutrition, and social isolation [27], although there is no apparent association with BMI [26, 28]. Currently the ORTO-15 questionnaire [29] is used to determine the presence of ON. Higher prevalence has been found in yoga instructors [30], dieticians, [31], nutrition students [32], and exercise science students [33] compared to the general population. The prevalence of ON in the general population was recently estimated to be less than 1% [34].

There are several reasons as to why ON does not currently appear in the DSM. For example, Varga et al. point out that there are psychometric limitations to the ORTO-15 questionnaire, such as issues with internal consistency, lack of standardisation, and cultural variation [24, 26, 35]. Furthermore, the high cut-off score may lead to false positives [36], arguably rendering the questionnaire unsuitable as a diagnostic tool. In addition, questions have been raised as to whether ON can simply be described as a subtype of another disorder, such as AN, OCD, or Avoidant/Restrictive Food Intake Disorder (ARFID) [37]. However, Dunn and Bratman [24] make a compelling argument that there is sufficient evidence to suggest ON is a distinct condition.

Currently the relationship between ON and social media use is unclear, although links between social media use and AN have been reported. Taken together, the reported negative effects of social media on psychological wellbeing, the role of Instagram in food sharing and negative social comparison, and the popularity of the healthy eating movement on Instagram suggests that there may be a positive relationship between ON and use of social media, and that Instagram may play a key role in the development and maintenance of disordered eating patterns. Accordingly, here we assess whether there is a link between Instagram use and symptoms of ON.

## Methods and materials

### Participants

Participants were recruited via not-paid-for advertisements on Instagram, Facebook and Twitter, as well as the blog “Plantbased Pixie”^2^ and the “Heath Bloggers Community” newsletter. Data were collected from 713 participants (23 males, 686 females, 4 other/prefer not to say. Mean age 24.96 ± 8.17 years). Non-female participants (*N* = 27) and participants for whom an ORTO-15 score could not be calculated due to missing data (*N* = 6) were excluded from the main analysis. We decided to concentrate on female participants as the *N* recruited for men was too low to provide adequate power for statistical analyses.

The final sample thus consisted of 680 females, age 18–75 years (mean = 24.70 ± 7.87) with an average healthy BMI (mean = 22.14 ± 3.89). 44.6% of participants lived in UK, 26.7% in the US, with the remainder living in an additional 40 countries. All participants gave their informed consent before starting the questionnaire and the study was approved by the UCL ethics committee.

### Survey

Our online survey was developed using Qualtrics (http://www.qualtrics.com). The questions we asked are detailed in the following.

#### Social media use

Participants were asked “Which social media channels do you use?” and could select multiple responses out of: Instagram, Facebook, Twitter, Pinterest, Google+, Tumblr, and LinkedIn. These are all content-based social media channels which have a ‘newsfeed’, and where other users can be added/followed. While Instagram and Pinterest are mainly image-based, Facebook, Twitter, Google+, and LinkedIn focus on text entries, with Tumblr having elements of both. Next, participants were asked about the amount of time spent on each channel they use: (1) “How often do you access [channel]?” with multiple-choice answers of “Less than once per month, 1–3 times per month, once a week, 2–3 times per week, 4–5 times per week, once a day, several times per day”, and (2) “On a typical day where you access [channel], how much time do you spend on it in total?” with multiple-choice answers of “Less than 15, 15–30, 31–60, 60+ min”. In addition, participants who indicated using Instagram were asked to rank-order Hu et al. [9] eight popular content types for the frequency with which they typically appear on their feed.

#### Dietary choices

Participants were asked which of 19 food types they included in their diet: red meat, white meat, processed meat, fish, dairy products, eggs, fruit, vegetables, whole-grains, refined grains, refined sugars, wheat, gluten, salt, oils, beans and pulses, processed/prepared foods, alcohol, and nightshades. Participants were also asked which label best described their diet: omnivore, vegetarian, vegan, pescatarian, paleo, plant-based, high-carb low-fat, raw vegan, other.

#### Orthorexia nervosa measure

The ORTO-15 questionnaire [29] was used to assess for orthorexic symptoms. ORTO-15 is a multiple-choice questionnaire consisting of 15 items with 4-point Likert-scale response options. A score of 1 is assigned to behaviours that most reflect orthorexic symptoms, with a score of 4 assigned to the most normal behaviours, meaning lower scores indicate higher levels of ON symptoms.

#### Demographics

Participants were asked their age (years), sex (male, female, other/prefer not to say), ethnicity, country of residence, height (cm or feet/inches), and weight (kg or lbs).

### Assessment and analysis

An ORTO-15 score was calculated for each participant. We used two cut-off scores due to disagreement in the literature over the appropriate cut-off point: less than 40 (following [28]), and less than 35 (following [35]). Participants’ social media time was converted into a score of approximate minutes per week spent on each channel (following [38]).

Height was converted to meters and weight to kg to calculate BMI. Only a small percentage of data was missing (3.82%), most of which was height and weight data.

Analyses were performed in Microsoft Excel 2016 and SPSS Version 24. Because data on social media use was non-parametric, Spearman’s rank order correlations were used to determine relationships between ORTO-15 score and social media use. Data on ORTO-15 scores was normally distributed, therefore, Pearson product-moment correlations were used to determine relationships between ORTO-15 scores and the variables age and BMI. Post-hoc analysis used Spearman’s rank order correlations to determine the relationship between ORTO-15 scores and both number of food types and number of social media channels.

## Results

### Sample characteristics

For a summary of demographic sample characteristics see Table 1. All but one participant used at least one social media channel (range = 0–7; mean = 3.55 ± 1.25), with Instagram being the most popular and Google+ the least popular (Table 2). 80% of participants who used Instagram ranked food as 1st or 2nd most frequent image category appearing on their Instagram feed.

**Table 1:** Demographic information for participants

Demographics	Participants (mean ± SD)
Age	24.70 ± 7.87
BMI	22.14 ± 3.89
Number of food items eaten (out of *n* = 19)	11.88 ± 3.67
Number of social media channels (out of *n* = 7)	3.55 ± 1.25
ORTO-15 score	34.33 ± 4.04

	Participants (%)
Diet	28.4% vegan, 24.7% omnivore, 12.2% vegetarian, 34.7% other
Country of residence	44.6% UK, 26.7% US, remaining from 40 other countries
Ethnicity	85.0% Caucasian, 6.0% Asian, 3.8% Hispanic, 5.2% other

**Table 2:** Prevalence of social media use, by % use of each channel, % visiting daily, and median time spent on each channel

	Instagram	Facebook	Twitter	Pinterest	Google+	Tumblr	LinkedIn
% using this channel	94.5%	88.1%	41.0%	54.9%	12.3%	20.7%	29.0%
% users visiting daily	95.0%	88.9%	56.7%	24.5%	31.8%	25.0%	7.7%
Median time users spend on this channel per day	15–30 min	15–30 min	<15 min	15–30 min	<15 min	15–30 min	<15 min

28.4% of participants reported following a vegan diet, 24.7% an omnivorous diet, and the remainder either a vegetarian, pescatarian, paleo, plant-based, high-carb low fat, raw vegan, or other diet. The mean number of food types eaten was 11.88 ± 3.67 of a possible 19.

The prevalence of ON in our sample was 90.6% with a cut-off score of <40, or 49.0% with a cut-off score of <35. This did not change amongst the sub-sample of Instagram users (*N* = 669; 90.4% <40, or 49.3% <35).

### Inferential Results

No relationship was found between ORTO-15 scores and number of food types eaten (*r* = −0.01; *p* = 0.87), number of social media channels used (*r* < 0.01; *p* = 0.99), age (*r* = −0.06; *p* = 0.13), or BMI (*r* = −0.03; *p* = 0.47). There were also no significant differences in ORTO-15 score between diet types as determined by one-way ANOVA [*F*(5,674) = 0.705, *p* = 0.620]. We, therefore, did not control for these variables in our correlational analyses.

**Fig. 1:**
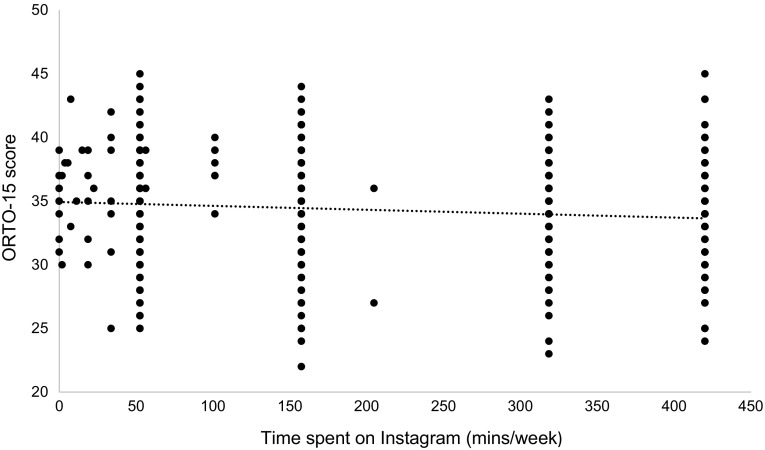
The negative relationship between time spent on Instagram and ORTO-15 score

As predicted, there was a negative correlation between ORTO-15 score and Instagram use (*r* = −0.10; *p* = 0.01) (Fig. 1). No significant relationship was found between ORTO-15 score and other social media use (Table 3), except Twitter which showed a small positive correlation (*r* = 0.12, *p* = 0.04).

**Table 3:** Correlations between time spent on each social media channel and ORTO-15 score

	Instagram	Facebook	Twitter	Pinterest	Google+	Tumblr	LinkedIn
ORTO-15	−0.10**	0.04	0.12*	−0.06	0.15	0.10	−0.04

*p < 0.05, **p < 0.01

To assess the robustness of the correlation between ORTO-15 and Instagram use when controlling for potentially confounding demographic variables, we additionally conducted linear regression analysis with ORTO-15 score as the outcome measure and Instagram use, age, BMI, ethnicity (dummy coded as white/non-white), and country of residence (dummy coded as UK/non-UK) as the predictor variables. The overall model was significant [*F*(5,648) = 9.58, *p* < 0.001)] with Instagram use (*β* = −0.12, *p* = 0.003) and country of residence (*β* = 0.23, *p* < 0.001) being significant predictors. The remaining variables were not significant (all *p* > 0.22).

## Discussion

This study investigated the relationship between social media use and ON. We found a significant relationship between symptoms of ON as measured by the ORTO-15 test and Instagram use, with higher Instagram use being associated with a greater tendency towards ON. No other social media channels were found to have this effect, although Twitter seemed to have a small protective association. While the effect size was small, the large population of social media users, now over 500 million on Instagram alone, means that this is a meaningful effect at population level.

There are several factors that likely contribute to the association between Instagram use and ON symptoms: First, the image-focused nature of Instagram, which plays to the picture-superiority effect—whereby images are more likely to be remembered than words [39]—and makes Instagram ideal for sharing food images. Second, social media allows for and encourages selective exposure, as users choose which accounts they wish to follow, and so are then continually exposed to the type of content these accounts produce. This limited exposure in turn may lead to users believing a behaviour is more prevalent or normal than is actually the case, and may lead to perceived social pressures to conform to such behaviours. Moreover, problematic behaviours may be continuously reinforced through image exposure and personal interactions on the platform. Third, there is likely a prevalence of eminence-based practice by which users with a large following may be perceived as authorities, allowing healthy eating ‘celebrities’ to influence large numbers of individuals by giving their followers a constant and curated feed of images portraying a certain diet or behaviour. Overall, these factors may explain why Instagram, an image-based social media, has been the platform of choice for the healthy eating community [3].

Although only Instagram was predicted to have an association with ON, exploratory analysis into other social media channels showed that Twitter had a positive association with ON symptoms. While purely exploratory, this is an interesting result that future work should seek to replicate. If correct, we speculate that this effect may be linked to the text-focused and strictly character-limited nature of twitter, which does not compliment a food-focused community as well as an image-based platform such as Instagram.

We believe this is the first study investigating a link between social media and ON, and it contributes to the growing body of literature on ON and its causes. The high prevalence of ON in the current study population is reflected in previous studies, where higher prevalence was found in yoga instructors (86%) [30], dieticians (41.9%) [31], nutrition students (35.9%) [32], exercise science students (84.5%) [33], and patients recovering from AN or bulimia nervosa (58%) [40] compared to the general population (<1%) [29, 34]. These are all populations who take an active interest in their health and body, in the same way that the healthy eating community on Instagram might be. When using the higher cut-off <40, the prevalence of ON in the current study population was higher than any other previously studied population, and occurred despite participants spending no more time on Instagram per day, on average, than the general population (15–30 vs. 21.2 min [8]). Even when using the lower cut-off <35, which may be more specific, the prevalence was still considerably higher than the general population. It is possible that we tapped a sub-group of Instagram users who are using it predominantly for food-related enquiries and who prescribe to the ‘healthy eating movement’.

The relationship between Instagram use and ON symptoms reported here has potential clinical implications, as there are overlaps between ON and AN [25], and ON has been found to be a frequent comorbidity with both AN and bulimia nervosa [40]. In fact, ON symptoms have been found to worsen following treatment for other eating disorders, suggesting that ON may be a compromise by which patients continue to exercise control over food and their body, although to a lesser degree than in AN. This is consistent with the finding that ON and BMI are not related [26, 28]. A lack of relationship between ON and type of diet further suggests that orthorexic behaviour is not limited to eliminating specific food groups.

### Limitations and further research

Participants were recruited through convenient non-random sampling, with the majority coming from the author’s Instagram account (83%), which is not representative of the general population. In addition, the research title (Health habits on social media) may have attracted individuals with an interest in health. Furthermore, BMI was calculated from self-report rather than measured, accordingly, we cannot rule out error and deliberate miss-reporting. Finally, we did not assess whether some participants followed a strict diet for medical reasons. While this is unlikely to affect the overall results, its omission may have introduced unnecessary noise.

Because a large proportion of our study sample used more than one social media channel, we cannot exclude the possibility of interactions between these channels. While this was not the focus of the current study, considering over half of online-active adults use more than one social media channel [6], such interactions may present an interesting avenue for future research. Future research may wish to further address these open questions. In the current study, we cannot establish a causal relationship between Instagram use and ON symptoms due to the correlational nature of our study, and future research may wish to assess possible causal link between these variables as well as possibly test intervention strategies for alleviating negative effects of Instagram use.

## Conclusion

The current findings suggest that within the study population, higher Instagram use was associated with stronger orthorexic symptoms. No other social media channel had this effect, although Twitter had a small positive association. Age, BMI, number of social media channels, and type of diet had no effect on orthorexia nervosa symptoms. Overall, these findings provide an initial indication of the role that modern social media may play in the onset and progression of eating disorders.

## Footnotes

1
https://top-hashtags.com/instagram/.

2
http://www.plantbased-pixie.com/my-survey-health-habits-on-social-media/.
